# Kallistatin reduces vascular senescence and aging by regulating microRNA‐34a‐SIRT1 pathway

**DOI:** 10.1111/acel.12615

**Published:** 2017-05-24

**Authors:** Youming Guo, Pengfei Li, Lin Gao, Jingmei Zhang, Zhirong Yang, Grant Bledsoe, Eugene Chang, Lee Chao, Julie Chao

**Affiliations:** ^1^ Department of Biochemistry and Molecular Biology Medical University of South Carolina Charleston South Carolina; ^2^ Division of Molecular Biology and Biochemistry School of Biological Sciences University of Missouri‐Kansas City Kansas City Missouri; ^3^ Department of Obstetrics and Gynecology College of Medicine Medical University of South Carolina Charleston South Carolina

**Keywords:** aging, oxidative stress, kallistatin, microRNA‐34a, sirtuin 1, vascular senescence

## Abstract

Kallistatin, an endogenous protein, protects against vascular injury by inhibiting oxidative stress and inflammation in hypertensive rats and enhancing the mobility and function of endothelial progenitor cells (EPCs). We aimed to determine the role and mechanism of kallistatin in vascular senescence and aging using cultured EPCs, streptozotocin (STZ)‐induced diabetic mice, and *Caenorhabditis elegans* (*C. elegans*). Human kallistatin significantly decreased TNF‐α‐induced cellular senescence in EPCs, as indicated by reduced senescence‐associated β‐galactosidase activity and plasminogen activator inhibitor‐1 expression, and elevated telomerase activity. Kallistatin blocked TNF‐α‐induced superoxide levels, NADPH oxidase activity, and microRNA‐21 (miR‐21) and p16^INK^ ^4a^ synthesis. Kallistatin prevented TNF‐α‐mediated inhibition of SIRT1, eNOS, and catalase, and directly stimulated the expression of these antioxidant enzymes. Moreover, kallistatin inhibited miR‐34a synthesis, whereas miR‐34a overexpression abolished kallistatin‐induced antioxidant gene expression and antisenescence activity. Kallistatin via its active site inhibited miR‐34a, and stimulated SIRT1 and eNOS synthesis in EPCs, which was abolished by genistein, indicating an event mediated by tyrosine kinase. Moreover, kallistatin administration attenuated STZ‐induced aortic senescence, oxidative stress, and miR‐34a and miR‐21 synthesis, and increased SIRT1, eNOS, and catalase levels in diabetic mice. Furthermore, kallistatin treatment reduced superoxide formation and prolonged wild‐type *C. elegans* lifespan under oxidative or heat stress, although kallistatin's protective effect was abolished in miR‐34 or sir‐2.1 (SIRT1 homolog) mutant *C. elegans*. Kallistatin inhibited miR‐34, but stimulated sir‐2.1 and sod‐3 synthesis in *C. elegans*. These *in vitro* and *in vivo* studies provide significant insights into the role and mechanism of kallistatin in vascular senescence and aging by regulating miR‐34a‐SIRT1 pathway.

## Introduction

Endothelial progenitor cells (EPCs) are a major contributor to vascular repair, and can be derived from bone marrow, circulating mononuclear cells, and cord blood (Lin *et al*., 2000; Reyes *et al*., 2002). Circulating EPCs are the most important cell population for the replenishment of damaged or senescent endothelial cells. Advanced aging is a major risk factor for suppressed EPC function (Williamson *et al*., 2012). Senescent or impaired EPCs contribute to endothelial dysfunction, which is a predictor of cardiovascular diseases (Heiss *et al*., 2005). Type 1 diabetes is one of cardiovascular‐associated diseases characterized by reduced EPC numbers and vascular repair (Loomans *et al*., 2004). Moreover, the nematode *Caenorhabditis elegans* (*C. elegans*) has a number of distinct advantages that are useful for understanding the molecular basis of organismal dysfunction underlying age‐related diseases. Due to its short life cycle (3.5 days) and conserved longevity genes from worm to human (Zhou *et al*., 2011), *C. elegans* is an ideal model for investigating the aging process. Consequently, EPCs, streptozotocin (STZ)‐induced diabetic mice, and *C. elegans* are valuable models for examining the mechanisms of vascular senescence and aging.

Oxidative stress is a key inducer of endothelial senescence, with the inflammatory cytokine TNF‐α being the main contributor to reactive oxygen species (ROS) production (Chen *et al*., 2008). Upregulation of antioxidant proteins, such as endothelial nitric oxide synthase (eNOS), sirtuin 1 (SIRT1), catalase, and manganese superoxide dismutase (MnSOD), has been shown to protect against oxidative stress‐mediated insults (Wassmann *et al*., 2004; Ota *et al*., 2010). eNOS maintains the redox state of endothelial cells and promotes vasorelaxation through NO production (Forstermann & Sessa, 2012). SIRT1 is a conservative longevity gene from yeast to human, and accounts for vascular homeostasis by activating many antioxidant enzymes, such as eNOS, catalase, and MnSOD, to diminish ROS (Kitada *et al*., 2016). Conversely, growing evidence has shown that microRNA‐34a (miR‐34a) is a senescence promoter, as it inhibits SIRT1 through a miR‐34a‐binding site within the 3′ UTR of SIRT1 (Zhao *et al*., 2010). Furthermore, miR‐21, a tumor inducer, is also involved in EPC senescence (Zhu *et al*., 2013). The antioxidant enzymes and pro‐senescence miRNAs underlie the molecular basis for endothelial senescence and aging‐associated diseases. Therefore, exploration of effective molecules or compounds that stimulate longevity gene expression and inhibit the effects of negative regulators may lead to a prospective enhancement of vascular integrity and lifespan.

Kallistatin was identified in human plasma as a tissue kallikrein‐binding protein and a serine proteinase inhibitor (Chao *et al*., 1986; Zhou *et al*., 1992). Kallistatin consists of two structural elements, an active site and a heparin‐binding site (Chen *et al*., 2000a,b, 2001), which regulate a wide spectrum of biological activities (Chao *et al*., 2016). The active site of kallistatin is crucial for inhibiting tissue kallikrein activity, and stimulating eNOS and SIRT1 expression (Chen *et al*., 2000a,b; Guo *et al*., 2015). Kallistatin's heparin‐binding domain is essential for antagonizing signaling pathways mediated by VEGF, TNF‐α, high‐mobility group box‐1 (HMGB1), and TGF‐β (Miao *et al*., 2003; Yin *et al*., 2010; Li *et al*., 2014; Guo *et al*., 2015). Kallistatin administration attenuates organ damage, inflammation, and oxidative stress associated with increased eNOS and NO levels in animal models of myocardial infarction and salt‐induced hypertension (Gao *et al*., 2008; Shen *et al*., 2008; Yin *et al*., 2010). Kallistatin, via NO stimulation, reduces TNF‐α‐induced superoxide production and NADPH oxidase activity in endothelial cells (Shen *et al*., 2010). Moreover, kallistatin prevents endothelial–mesenchymal transition (EndMT) by inhibiting TGF‐β‐induced miR‐21, and increasing SIRT1 synthesis in endothelial cells (Guo *et al*., 2015). Importantly, kallistatin treatment increases circulating EPC number and reduces aortic oxidative stress in hypertensive rats, whereas kallistatin deficiency decreases EPC levels and exacerbates oxidative vascular injury (Liu *et al*., 2012; Gao *et al*., 2014). In addition, kallistatin promotes vascular repair by enhancing the viability, migration, and function of EPCs (Gao *et al*., 2014). These findings led us to investigate the potential role of kallistatin in vascular senescence and aging, using both *in vitro* and *in vivo* models.

## Results

### Kallistatin inhibits TNF‐α‐induced cellular senescence in EPCs

**Figure 1:**
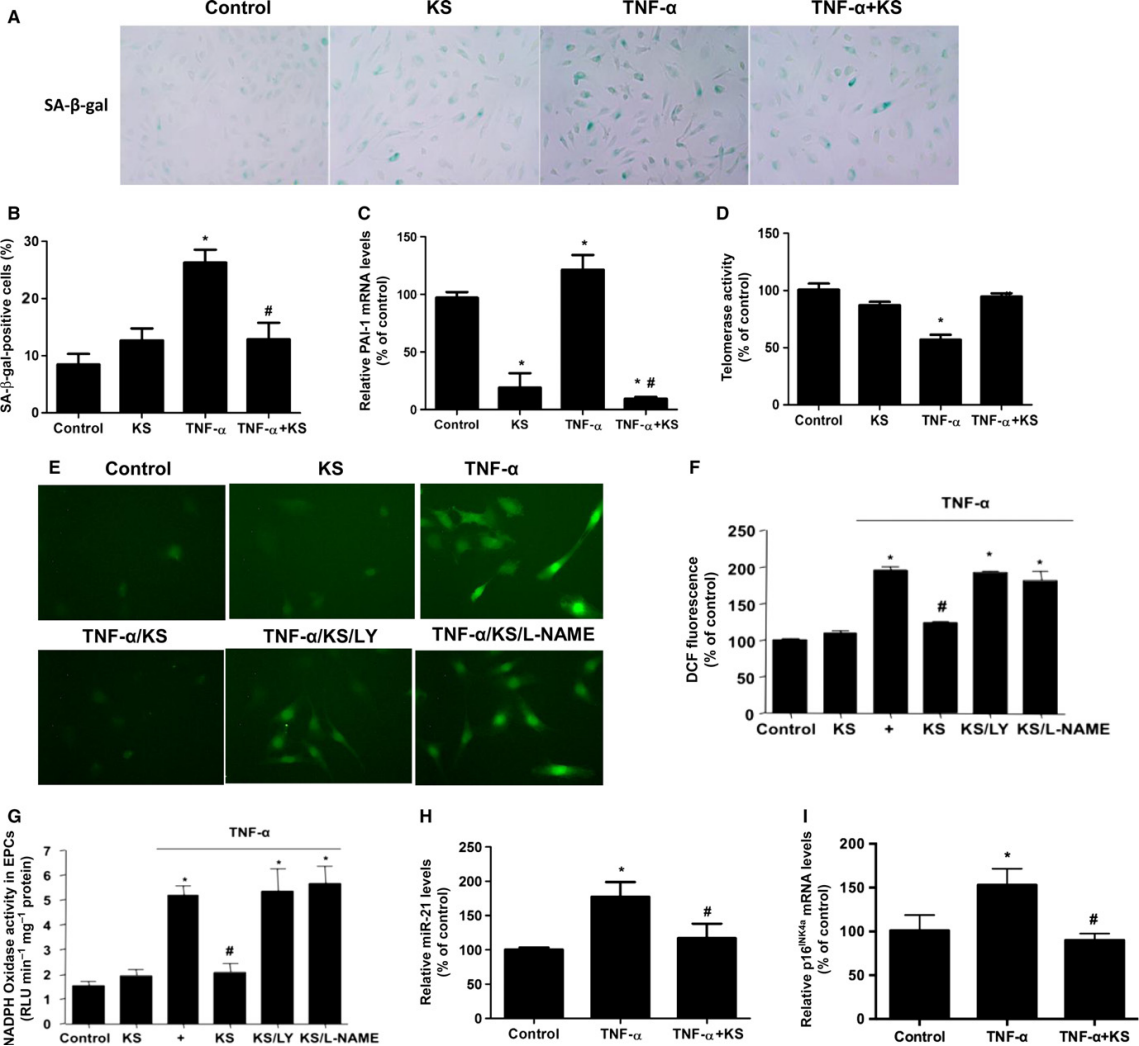
Kallistatin (KS) inhibits TNF‐α‐induced endothelial senescence and oxidative stress in EPCs. (A) Representative images of senescence‐associated β‐gal staining in EPCs are shown. (B) Quantification analysis of positive SA‐β‐gal staining cell number from three biological replicates. For each replicate, at least 10 random microscopic fields were counted. (C) Real‐time PCR analysis of PAI‐1. (D) Telomerase activity after 24 h of TNF‐α treatment. (E) Representative fluorescent images of ROS formation in EPCs were shown using ROS probe DCFH‐DA. EPCs were pre‐incubated with PI3K inhibitor LY294002 or NOS inhibitor L‐NAME for 30 min in prior to KS treatment. (F) Quantification analysis of ROS production. (G) NADPH oxidase activity determined by lucigenin chemiluminescence assay. (H) Real‐time PCR analysis of p16^INK^ ^4a^, (I) miR‐21 in TNF‐α treated EPCs with or without KS addition. *n* = 3. Values are expressed as mean ± SE; *P <* *0.05 vs. control, ^#^ *P *<* *0.05 vs. TNF‐α group.

To identify the effect of kallistatin on cellular senescence in cultured EPCs, we evaluated senescence markers, including SA‐β‐gal activity, plasminogen activator inhibitor‐1 (PAI‐1) synthesis, and telomerase activity. Exposure of EPCs to TNF‐α for 6 days markedly increased SA‐β‐gal‐positive cell numbers compared with the control group, whereas pre‐incubation with purified human kallistatin significantly reduced TNF‐α‐induced SA‐β‐gal‐positive cells. Kallistatin alone had no effect on EPC senescence (Fig. 1A). Quantitative analysis of SA‐β‐gal staining confirmed these results (Fig. 1B). TNF‐α increased PAI‐1 synthesis, whereas kallistatin significantly suppressed PAI‐1 expression with or without TNF‐α treatment (Fig. 1C). Moreover, kallistatin prevented TNF‐α‐mediated suppression of telomerase activity in EPCs (Fig. 1D). These results indicate that kallistatin is capable of blocking TNF‐α‐induced EPC senescence.

### Kallistatin inhibits TNF‐α‐induced oxidative stress, and miR‐21 and p16^INK4a^ synthesis

To further determine kallistatin's effect on senescence‐associated oxidative stress in EPCs, fluorescence probe DCFH‐DA was used to detect cellular ROS formation. Representative images show that kallistatin inhibited TNF‐α‐induced accumulation of cellular ROS in EPCs, and kallistatin's effect was abolished by pretreatment with the PI3K inhibitor LY294002 or the NOS inhibitor L‐NAME (Fig. 1E). Quantitative analysis verified kallistatin's inhibitory effect on ROS formation (Fig. 1F). Likewise, kallistatin suppressed TNF‐α‐induced NADPH oxidase activity, which was again abolished by LY294002 or L‐NAME (Fig. 1G). Kallistatin alone had no effect on oxidative stress (Fig. 1E–G). These results indicate that kallistatin inhibits TNF‐α‐induced oxidative stress via activation of the PI3K‐Akt‐eNOS signaling pathway. Kallistatin also antagonized TNF‐α‐induced miR‐21 synthesis (Fig. 1H). Moreover, kallistatin markedly reduced the expression of p16^INK4a^, a cyclin‐dependent kinase inhibitor known to be a senescence‐associated inducer of cell cycle arrest (Fig. 1I). Collectively, these results indicate that kallistatin inhibits TNF‐α‐induced oxidative stress, and miR‐21 and p16^INK4a^ synthesis.

### Kallistatin prevents TNF‐α‐mediated inhibition of SIRT1, eNOS, and catalase and stimulates antioxidant gene expression

**Figure 2:**
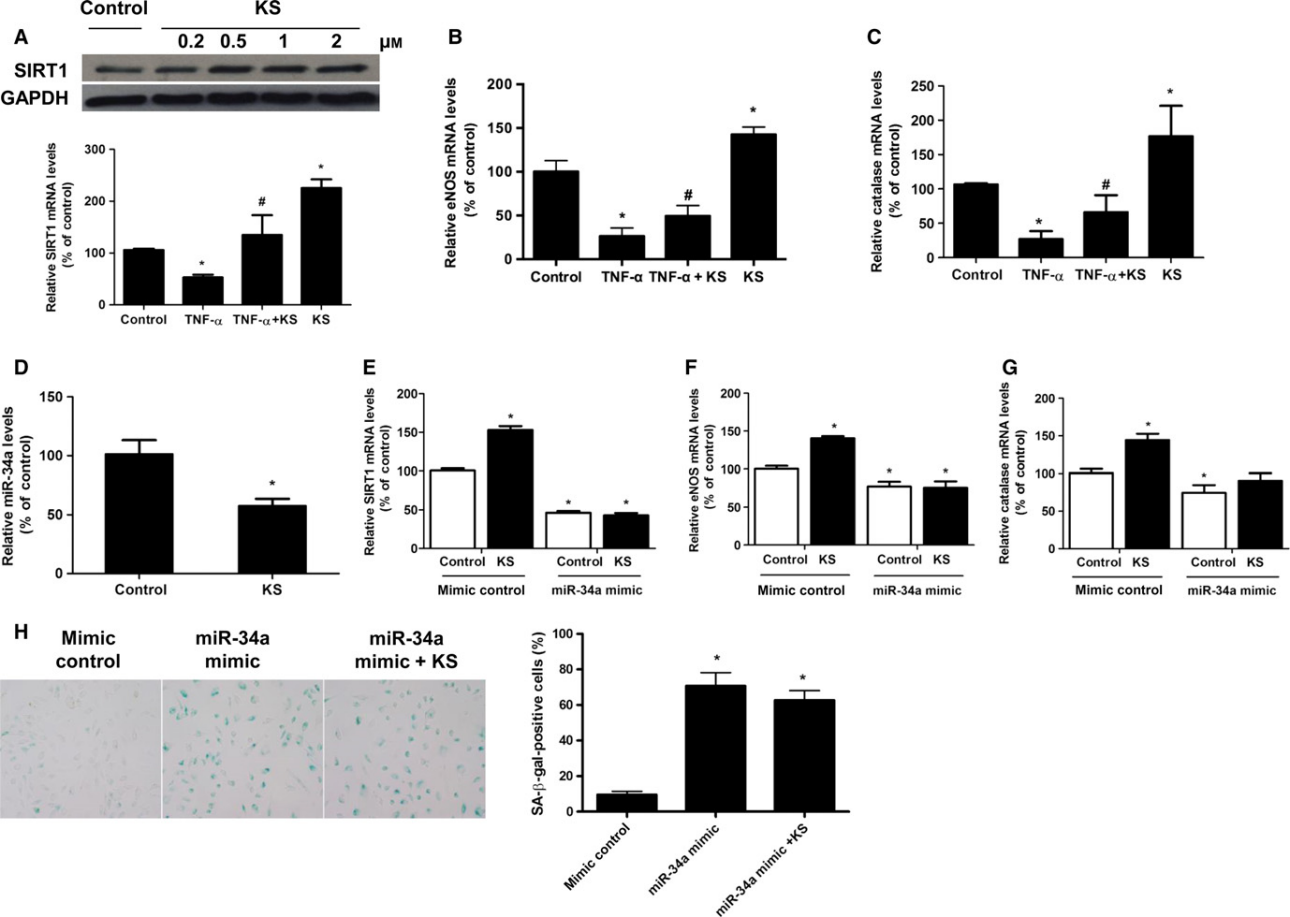
Kallistatin (KS) stimulates SIRT1, eNOS, and catalase synthesis through inhibition of miR‐34a in EPCs. (A) KS's effects on mRNA and protein levels of SIRT1, (B) mRNA levels of eNOS, (C) mRNA levels of catalase, and (D) miR‐34a synthesis. (E) SIRT1 expression upon KS treatment in mimic control and miR‐34a‐overexpressing EPCs. Mimic control and miR‐34a mimic were, respectively, transferred to EPCs for 12 h in prior to KS treatment. (F) eNOS and (G) catalase expression upon KS treatment in mimic control and miR‐34a‐overexpressing EPCs. (H) Representative images of positive SA‐β‐gal staining in mimic control and miR‐34a‐overexpressing EPCs, and quantification analysis from three independent experiments are shown. *n* = 3 for A–D, *P <* *0.05 vs. control, ^#^ *P *<* *0.05 vs. TNF‐α group. *n* = 6 for E‐H, *P <* *0.05 vs. mimic control. Values are expressed as mean ± SE.

We next determined the effect of kallistatin on the antioxidant genes of SIRT1, eNOS, and catalase. Kallistatin treatment increased SIRT1 protein levels, as determined by Western blot (Fig. 2A, top panel). Moreover, kallistatin reversed TNF‐α‐mediated inhibition of SIRT1, eNOS, and catalase expression (Fig. 2A–C). Additionally, kallistatin alone stimulated the synthesis of these antioxidant enzymes (Fig. 2A–C). Therefore, kallistatin not only prevented TNF‐α‐mediated inhibition of eNOS, SIRT1, and catalase, but also increased the expression levels of antioxidant enzymes.

### Kallistatin stimulates antioxidant gene expression through miR‐34a inhibition

As miR‐34a is a pro‐senescence gene, we examined kallistatin's effect on miR‐34a. Our results showed that kallistatin markedly inhibited miR‐34a synthesis in EPCs (Fig. 2D). To further determine the role of miR‐34a in kallistatin‐mediated stimulation of antioxidant genes, miR‐34a was overexpressed by transfection of miR‐34a mimic to EPCs. miR‐34a overexpression suppressed SIRT1, eNOS, and catalase expression compared to the mimic control group, and also abolished kallistatin's stimulatory effect on these antioxidant enzymes (Fig. 2E–G). Moreover, miR‐34a overexpression abolished kallistatin's antisenescence effect, evidenced by SA‐β‐gal staining (Fig. 2H). These results indicate that kallistatin, by inhibiting miR‐34a synthesis, prevents miR‐34a‐mediated inhibition of SIRT1 and eNOS expression to alleviate EPC senescence.

### Kallistatin's active site is essential for stimulating antioxidant gene expression: role of tyrosine kinase

**Figure 3:**
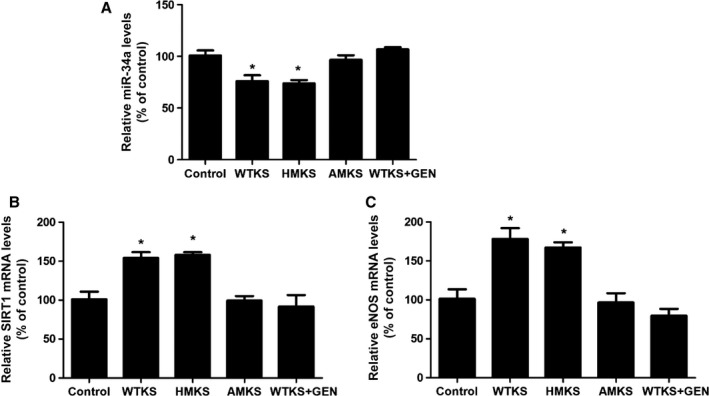
The active site of kallistatin (KS) is essential for modulating miR‐34a, eNOS, and SIRT1 synthesis through interacting with a tyrosine kinase in EPCs. EPCs were pre‐incubated with or without a tyrosine kinase inhibitor genistein (GEN) for 30 min, followed by treatment with wild‐type KS (WTKS), heparin‐binding site mutant KS (HMKS), or active site mutant KS (AMKS) for additional 24 h. The changes in (A) miR‐34a levels, (B) SIRT1 mRNA levels, and (C) eNOS mRNA levels were measured by Q‐PCR. *n* = 3. Values are expressed as mean ± SE; *P <* *0.05 vs. control.

To determine which functional domain of kallistatin is essential for the regulation of miR‐34a‐SIRT1 pathway, three types of kallistatin, including wild‐type kallistatin, heparin‐binding site mutant kallistatin, and active site mutant kallistatin, were used. We found that both wild‐type kallistatin and heparin‐binding site mutant kallistatin effectively inhibited miR‐34a synthesis and elevated SIRT1 and eNOS mRNA levels in EPCs, but active site mutant kallistatin had no such effects (Fig. 3A–C). The results indicate that kallistatin's active site is essential for modulating the expression of miR‐34a, SIRT1, and eNOS in EPCs. Moreover, genistein, a tyrosine kinase inhibitor, blocked wild‐type kallistatin's effect in modulating miR‐34a, SIRT1, and eNOS synthesis (Fig. 3A–C), implicating the involvement of a tyrosine kinase. Thus, kallistatin's active site is critical for downregulation of miR‐34a and upregulation of SIRT1 and eNOS.

### Kallistatin attenuates aortic senescence, oxidative stress, and miR‐34a and miR‐21 synthesis, but increases SIRT1, eNOS, and catalase levels in diabetic mice

**Figure 4:**
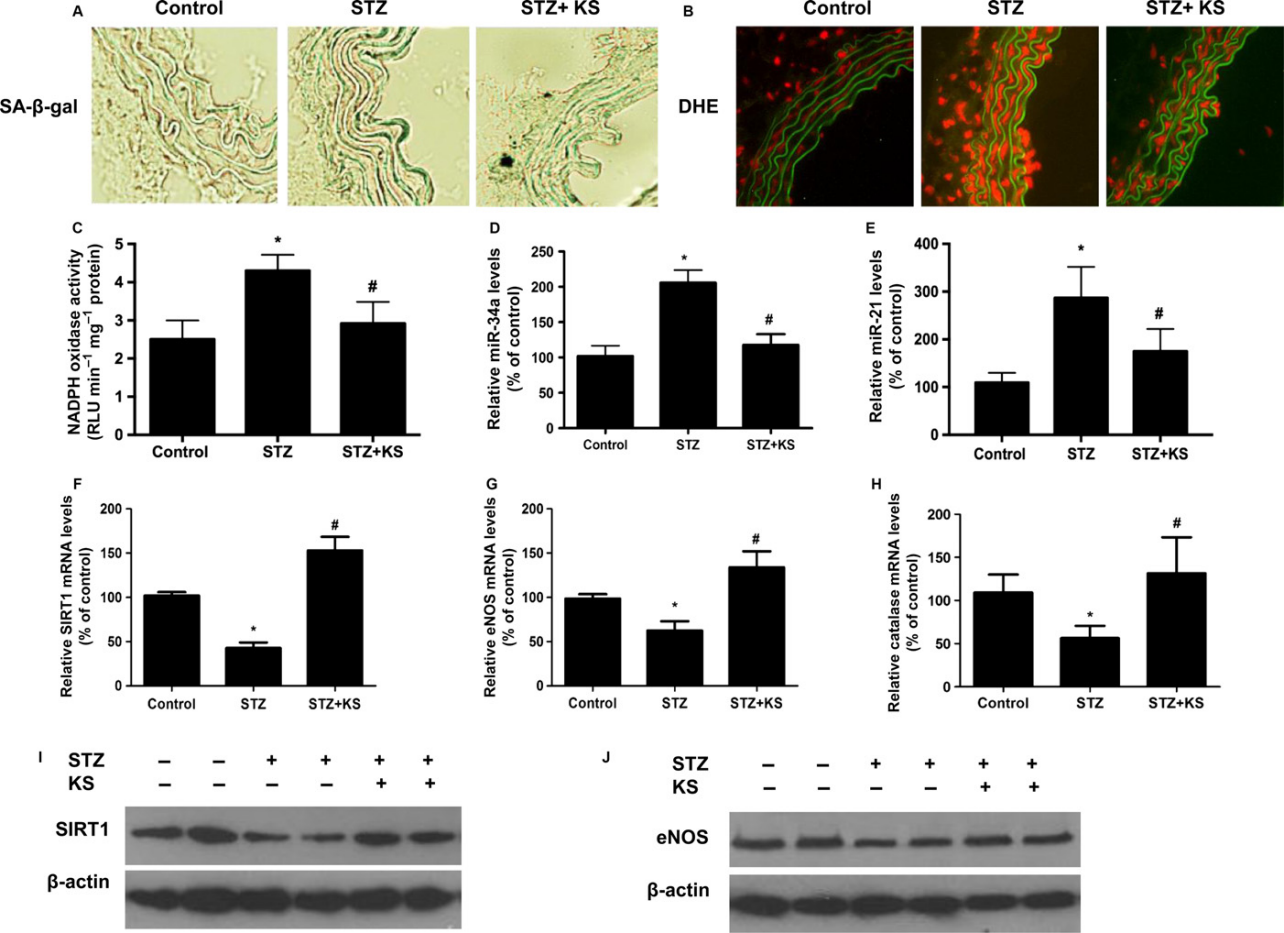
Kallistatin (KS) attenuates vascular aging and superoxide production, and miR‐21 and miR‐34a synthesis, and stimulates antioxidant gene expression in the aortas of diabetic mice. (A) Representative images of SA‐β‐gal staining in thoracic aortas from control mice, STZ‐induced diabetic mice, and KS‐treated diabetic mice (*n* = 6 each group). (B) Representative images of superoxide formation labeled by red fluorescence dye hydroethidine (DHE) in the aortas of control mice, diabetic mice, and KS‐treated diabetic mice (*n* = 6 each group). (C) NADPH oxidase activity in aortas of control mice, diabetic mice, and KS‐treated diabetic mice (*n* = 6 each group). (D) KS's effects on the synthesis of miR‐34a, (E) miR‐21, (F) SIRT1, (G) eNOS, and (H) catalase in the aortas of control mice and diabetic mice determined by Q‐PCR. (I) Representative Western blots of SIRT1 and (J) eNOS in the aortas of control mice, diabetic mice, and KS‐treated diabetic mice. Values are expressed as mean ± SE; *P <* *0.05 vs. control group; ^*#*^ *P *<* *0.05 vs. STZ group.

We further determined kallistatin's *in vivo* effect on vascular senescence and oxidative stress in aortas of STZ‐induced diabetic mice. Aortic senescence, identified by SA‐β‐gal staining, was increased in diabetic mice compared to control mice, while kallistatin treatment prevented STZ‐induced effect (Fig. 4A). Moreover, higher levels of superoxide formation and NADPH oxidase activity were observed in the aortas of STZ‐induced diabetic mice, but this observation was reversed by kallistatin administration (Fig. 4B,C). Furthermore, STZ induced a significant increase of miR‐34a and miR‐21 synthesis, as well as reduced SIRT1, eNOS, and catalase mRNA levels in aortas of diabetic mice compared to control mice, while kallistatin administration reversed STZ‐mediated effect (Fig. 4D–H). Consistently, aortic SIRT1 and eNOS protein levels were markedly reduced in diabetic mice compared to control mice, but were restored by kallistatin administration, as determined by Western blot (Fig. 4I,J). Consistent with the results derived from cultured EPCs, kallistatin treatment protects against vascular aging and oxidative stress in diabetic mice by decreasing miR‐34a and miR‐21 synthesis and increasing SIRT1, eNOS, and catalase levels.

### Kallistatin enhances the lifespan of wild‐type *C. elegans* under oxidative or heat stress, but has no effect in miR‐34 or sir‐2.1 (SIRT1) mutant worms

**Figure 5:**
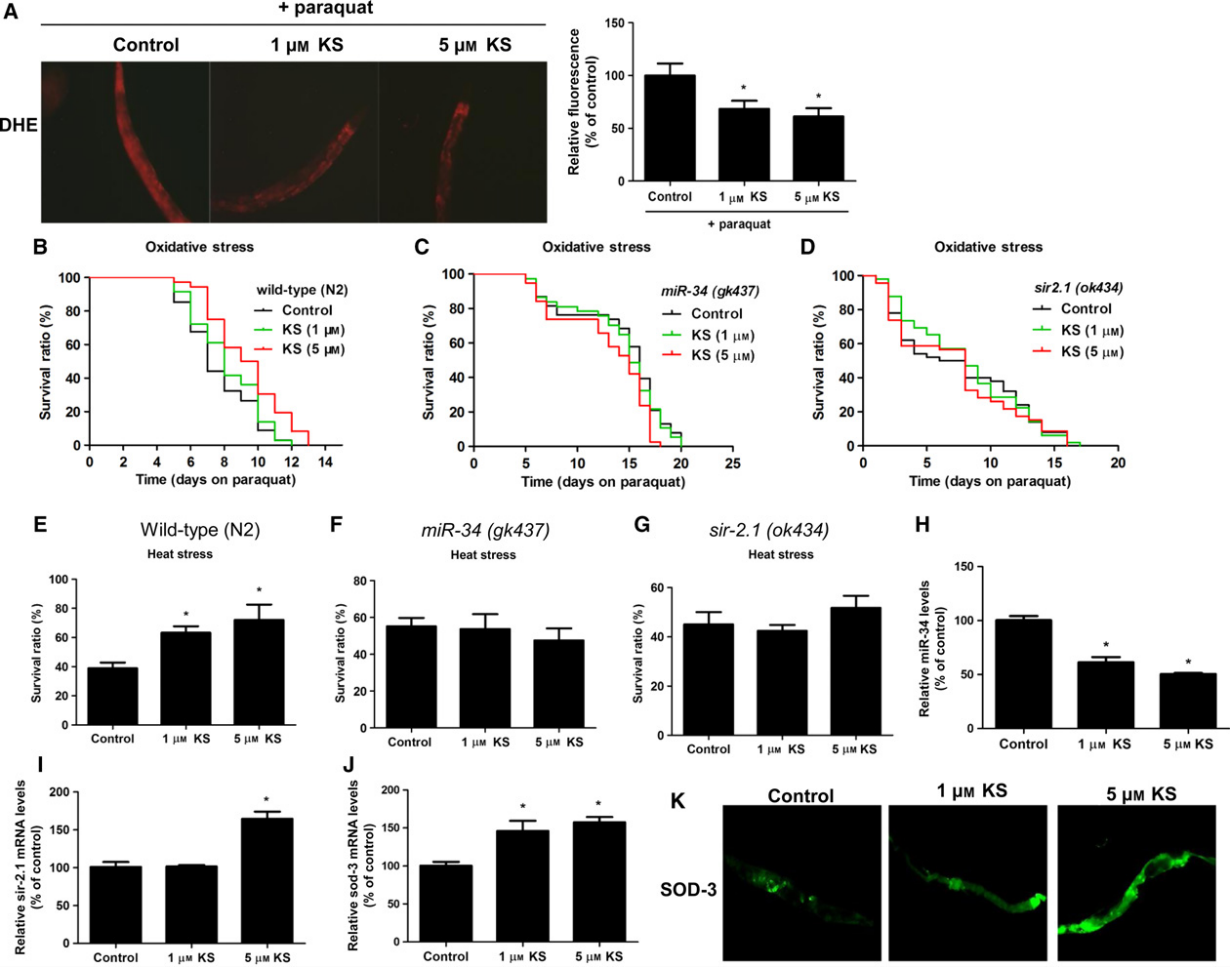
Kallistatin (KS) enhances the *Caenorhabditis elegans* survival under heat or oxidative stress by modulating miR‐34‐sir‐2.1 pathway. (A) Representative images of *in vivo* superoxide formation labeled with fluorescence dye DHE in wild‐type *C. elegans*, and densitometric analysis of *in situ* ROS generation were shown. (B) Survival curve of wild‐type (N2) strain, (C) miR‐34 mutant, and (D) sir‐2.1 mutant. *Caenorhabditis elegans* were treated with or without KS for 48 h, and subjected to paraquat‐induced oxidative injury at 25 °C. (E) Average survival ratios of wild‐type (N2) worm, (F) miR‐34 mutant, and (G) sir‐2.1 mutant. Worms were treated with or without KS for 48 h, and cultured at 35 °C for 6 h for heat shock. (H) KS's effect on miR‐34 synthesis. (I) sir‐2.1 mRNA and (J) sod‐3 mRNA levels in wild‐type *C. elegans* were determined by Q‐PCR. (K) Representative images of SOD‐3::GFP *C. elegans* treated with KS for 24 h. *n* = 3. Values are expressed as mean ± SE; *P <* *0.05 vs. control.

As stress conditions impair *C. elegans* longevity and induce premature senescence, we investigated the effect of kallistatin on *C. elegans* under oxidative or thermal stress. Paraquat was used to induce oxidative damage in three strains of *C. elegans*, including wild‐type (N2), miR‐34 mutant, and sir‐2.1 (SIRT1 analogue) mutant worms. Representative images showed that kallistatin (1 or 5 μm) markedly reduced paraquat‐induced superoxide formation in *C. elegans*, as identified by red fluorescence probe DHE, and the result was confirmed by quantitative analysis (Fig. 5A). Moreover, the mean lifespan of wild‐type *C. elegans* was 13.3 ± 0.9 days under normal condition at 25 °C, but was shortened to 7.7 ± 0.3 days under paraquat‐induced oxidative stress. Pretreatment with 1 or 5 μm kallistatin reduced the sensitivity to paraquat and increased mean lifespan of *C. elegan*s by 5.8% or 20.5% (8.2 ± 0.3 or 9.3 ± 0.4 days), respectively (Fig. 5B). However, kallistatin treatment had no effect on the lifespan of miR‐34 or sir‐2.1 mutant worms under oxidative stress (Fig. 5C,D). The results indicate that miR‐34 and sir‐2.1 are essential for kallistatin‐mediated *C. elegans* longevity under oxidative stress.

Kallistatin significantly increased *C. elegans* thermo‐tolerance at 35 °C. Wild‐type worm survival rate was 38.9 ± 3.9% under thermal condition, but kallistatin treatment at 1 and 5 μm markedly enhanced worm survival to 63.3 ± 4.4% and 72.1 ± 10.5%, respectively (Fig. 5E). However, kallistatin (1 or 5 μm) had no effect on the survival of miR‐34 mutant or sir‐2.1 mutant worms at 35 °C (Fig. 5F,G). Similar to oxidative stress, these results confirm that kallistatin promotes *C. elegans* survival under heat stress via regulating miR‐34 and sir‐2.1. Moreover, kallistatin treatment inhibited miR‐34, but increased sir‐2.1 and sod‐3 (MnSOD analogue) synthesis (Fig. 5H–J). Likewise, SOD‐3 protein levels were elevated by kallistatin treatment, as evidenced using SOD‐3::GFP co‐expression *C. elegans* as a sod‐3 indicator (Fig. 5K). Collectively, kallistatin inhibits miR‐34 synthesis and stimulates sir‐2.1 and sod‐3 levels in *C. elegans,* and the regulatory mechanisms parallel those observed in cultured EPCs and in aortas of diabetic mice.

## Discussion

**Figure 6:**
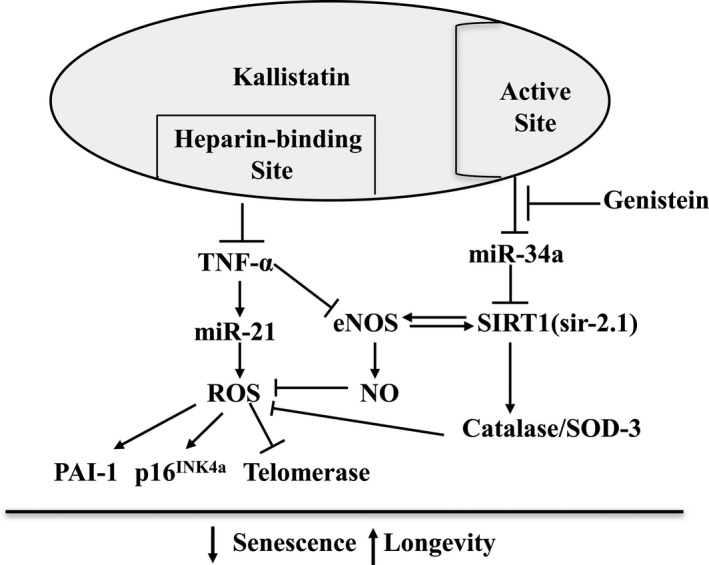
Proposed mechanisms by which kallistatin inhibits endothelial senescence and aging. Kallistatin via its heparin‐binding site antagonizes TNF‐α‐mediated miR‐21 synthesis, ROS formation, PAI‐1 and p16^INK^ ^4a^ expression, and telomerase activity in EPCs. Kallistatin via its active site prevents miR‐34a‐mediated inhibition of antioxidant genes SIRT1, eNOS, and catalase and SOD‐3. The tyrosine kinase inhibitor, genistein, blocks kallistatin's ability to modulate miR‐34a and antioxidant gene expression.

This study demonstrates a novel role of kallistatin in vascular senescence and aging using cultured EPCs, STZ‐induced diabetic mice, and *C. elegans*. Recombinant human kallistatin treatment significantly reduced EPC senescence by blocking TNF‐α‐induced oxidative stress, and decreased the senescence markers, PAI‐1, miR‐21, and p16^INK4a^, but increased telomerase activity. Kallistatin via its active site inhibited miR‐34a and stimulated SIRT1, eNOS, and catalase synthesis, whereas overexpression of miR‐34a abolished kallistatin's effects on these antioxidant enzymes and antisenescence action. Moreover, kallistatin suppressed miR‐34a and stimulated SIRT1 and eNOS expression, through interaction with cell surface tyrosine kinase in EPCs. Likewise, kallistatin administration in STZ‐induced diabetic mice alleviated aortic senescence and oxidative stress associated with reduced miR‐34a and miR‐21 synthesis and increased SIRT1, eNOS, and catalase levels. Moreover, kallistatin prolonged wild‐type *C. elegans* lifespan under heat or oxidative stress conditions by inhibiting miR‐34, and stimulating sir‐2.1 (SIRT1). These *in vitro* and *in vivo* studies support the view that kallistatin protects against vascular senescence and aging by preventing miR‐34a‐mediated inhibition of SIRT1 and eNOS, thus inhibiting oxidative stress. The signaling mechanisms of kallistatin's antisenescence actions are depicted in Fig. 6.

Oxidative stress is the leading cause of endothelial dysfunction and senescence. The inflammatory factor TNF‐α has the ability to potentiate ROS generation by activating NADPH oxidase on the endothelial cell surface (Yoshida & Tsunawaki, 2008). Conversely, eNOS reduces intracellular superoxide levels through NO formation, which inhibits NADPH oxidase activity (Fujii *et al*., 1997). In this study, we demonstrated that kallistatin inhibits TNF‐α‐induced oxidative stress via activation of the PI3K‐Akt‐eNOS signaling pathway, as kallistatin's effect is blocked by LY and L‐NAME. Likewise, our previous report showed that kallistatin antagonizes TNF‐α‐mediated apoptosis through increased Akt‐eNOS phosphorylation in endothelial cells (Shen *et al*., 2010). Kallistatin also promotes the proliferation and migration of EPCs via activation of Akt‐eNOS signaling pathway (Gao *et al*., 2014). Thus, it is likely that kallistatin antagonizes TNF‐α‐induced senescence by activation of Akt and eNOS phosphorylation in EPCs. In addition, miR‐21 modulates ROS production in several cell types (Zhang *et al*., 2012; Jiang *et al*., 2014), and kallistatin inhibits TGF‐β induced miR‐21 synthesis in endothelial cells (Guo *et al*., 2015). Our current data showed that kallistatin also blocked TNF‐α‐mediated miR‐21 expression and ROS production in EPCs. Kallistatin's vasoprotective effect has been shown in animal models of salt‐induced hypertension, renal injury, and chronic myocardial infarction through inhibiting oxidative stress (Gao *et al*., 2008; Shen *et al*., 2008, 2010). Consistently, our current study showed that kallistatin attenuated vascular aging and oxidative stress in conjunction with increased antioxidant protein levels in the aortas of STZ‐induced diabetic mice. Likewise, kallistatin reduced paraquat‐induced superoxide levels, and improved *C. elegans* lifespan under oxidative stress by stimulating sir‐2.1 (SIRT1) synthesis. Taken together, these *in vitro* and *in vivo* studies indicate that kallistatin, via inhibiting oxidative stress, protects against vascular senescence and aging.

The longevity factor SIRT1 is a NAD^+^‐dependent histone deacetylase, and high levels of SIRT1 were shown to inhibit oxidative stress and DNA damage (Alcendor *et al*., 2007). SIRT1 activates antioxidant enzymes, including catalase and MnSOD, by stimulating the FOXO transcription factors (Brunet *et al*., 2004). Through a positive feedback loop, SIRT1 activates eNOS, which in turn activates SIRT1 (Ota *et al*., 2010). Importantly, SIRT1 is highly expressed during angiogenesis in endothelial cells, and disrupting SIRT1 abrogates vascular endothelial homeostasis and remodeling (Potente *et al*., 2007). Our results indicate that kallistatin treatment stimulated the expression of SIRT1 and its downstream antioxidant enzymes eNOS and catalase, as well as antagonized TNF‐α‐mediated inhibition of SIRT1, eNOS, and catalase synthesis in cultured EPCs. Similarly, kallistatin elevated the levels of these antioxidant enzymes in the aortas of STZ‐induced diabetic mice. In *C. elegans*, SIRT1 homolog sir‐2.1 and MnSOD homolog sod‐3 were also increased upon kallistatin treatment, whereas sir‐2.1 mutant abolished kallistatin‐mediated stress resistance. Collectively, these combined results reveal a critical role of SIRT1 in mediating kallistatin's effect on oxidative stress and vascular aging.

The role of miR‐34a in regulating cell senescence, differentiation, and vitality has been extensively highlighted in a wide variety of cells (Tazawa *et al*., 2007; Zhao *et al*., 2010). miR‐34a triggers senescence partly through genetic inhibition of SIRT1 in EPCs (Zhao *et al*., 2010). This observation inspired us to explore the potential role of kallistatin in modulating miR‐34a‐dependent SIRT1 expression in EPCs. Our results showed that kallistatin significantly suppressed miR‐34a synthesis, but stimulated eNOS, SIRT1, and catalase expression in EPCs and aortas of diabetic mice. Intriguingly, overexpression of miR‐34a by transfection of miR‐34a mimic abolished kallistatin‐induced antioxidant gene expression. Moreover, miR‐34a mimic markedly induced EPC senescence; however, kallistatin had no effect on miR‐34a‐induced EPC senescence. These findings indicate that kallistatin's antisenescence effect is mediated by suppressing miR‐34a synthesis in EPCs. Furthermore, genistein, a tyrosine kinase inhibitor, blocked kallistatin's effects on the expression of miR‐34a, eNOS, and SIRT1. The results reveal that kallistatin, through interacting with tyrosine kinase, downregulates miR‐34a leading to reduced vascular senescence.

Kallistatin via its two structural elements regulates differential signaling pathways (Chao *et al*., 2016). Kallistatin's heparin‐binding site is essential for competing with the binding of VEGF, TNF‐α, HMGB1, and TGF‐β to their respective receptors by binding to cell surface heparan sulfate proteoglycans (Miao *et al*., 2003; Yin *et al*., 2010; Li *et al*., 2014; Guo *et al*., 2015). Kallistatin via its heparin‐binding site antagonized TNF‐α‐induced NF‐κB activation and thus inflammatory gene expression (Yin *et al*., 2010). Our current finding indicates that kallistatin also antagonized TNF‐α‐induced senescence and oxidative stress in EPCs. Kallistatin blocks TNF‐α‐induced endothelial senescence mediators, miR‐21 and p16^INK4a^. Likewise, kallistatin markedly downregulated TNF‐α‐induced expression of PAI‐1, a pro‐atherogenic factor and an endothelial aging marker. Furthermore, kallistatin reversed TNF‐α‐mediated inhibition of telomerase, which is responsible for maintaining telomere length and cellular integrity. Consistently, our recent report showed that circulatory kallistatin levels are positively associated with leukocyte telomere length in humans (Zhu *et al*., 2016), indicating a potential role of kallistatin in maintaining telomere length. Therefore, kallistatin via its heparin‐binding site blocks TNF‐α‐mediated effects in EPCs. Meanwhile, kallistatin's active site is essential for stimulating eNOS and SIRT1 in endothelial cells (Guo *et al*., 2015). In this study, we showed that kallistatin through its active site inhibited miR‐34a synthesis and stimulated eNOS and SITR1 expression in EPCs. Therefore, kallistatin, through its two functional domains, displays antisenescence actions by blocking TNF‐α‐induced effects, and preventing miR‐34a‐mediated inhibition of SIRT1 and eNOS.

The STZ‐induced diabetic mouse is a popular model for studying vascular injury and aging. Kallistatin levels are markedly reduced in vitreous fluids of patients with diabetic retinopathy and in the retinas in STZ‐induced diabetic rats (Ma *et al*., 1996; Hatcher *et al*., 1997). Decreased kallistatin levels are also observed in the kidney of STZ‐induced diabetic mice and in TNF‐α‐induced senescent human EPCs (unpublished data). Moreover, endogenous kallistatin plays a protective role in multi‐organ function, as depletion of kallistatin by neutralizing antibody injection augmented cardiovascular and renal damage, and increased oxidative stress, inflammation, and endothelial cell loss in hypertensive rats (Liu *et al*., 2012). Furthermore, kallistatin treatment exerts renoprotective effects in diabetic nephropathy by suppressing ROS and inflammatory gene expression in mice (Yiu *et al*., 2016). Accumulating evidence indicates that kallistatin acts as a potent antioxidant and anti‐inflammatory agent in cultured cells and animal models (Gao *et al*., 2008; Shen *et al*., 2010; Yin *et al*., 2010; Li *et al*., 2014; Guo *et al*., 2015). As oxidative endothelial injury and aging are observed in diabetes, kallistatin may have a role in protection against vascular damage in diabetic disease. Indeed, our current study showed that kallistatin treatment in STZ‐induced diabetic mice attenuated aortic senescence and superoxide formation, in association with reduced miR‐34a and miR‐21, and increased SIRT1, eNOS, and catalase synthesis. The findings in diabetic mice support the concept that kallistatin plays a protective role in vascular injury and aging by regulating miR‐34a, SIRT1, and eNOS synthesis.

*Caenorhabditis elegans* is a prominent model for aging study, as pathways mediating longevity and metabolism are conserved from *C. elegans* to mammals. Heat or oxidative stress induces elevated levels of ROS and cellular damage in *C. elegans*, leading to accelerated aging (Rodriguez *et al*., 2013). In this study, we demonstrated that kallistatin treatment improved *C. elegans* lifespan or survival under oxidative stress or heat conditions. Previous studies showed that miR‐34 loss‐of‐function mutation or sir‐2.1 (SIRT1 homolog) overexpression in *C. elegans* markedly delayed the age‐related physiological decline and prolonged lifespan in response to stress conditions (Tissenbaum & Guarente, 2001; Yang *et al*., 2013). Herein, we showed that kallistatin treatment inhibited miR‐34 and stimulated sir‐2.1 and sod‐3 synthesis in wild‐type *C. elegans*. Kallistatin's protective effect in the longevity of wild‐type *C. elegans* was abolished in miR‐34 or sir‐2.1 mutant *C. elegans* under stress conditions. Consistent with the findings in EPCs and diabetic mice, these results further verify that kallistatin's anti‐aging effect is dependent on miR‐34 inhibition and sir‐2.1 (SIRT1) activation. Collectively, these findings provide significant insights regarding the mechanism of kallistatin in regulating the aging process via miR‐34a‐SIRT1 pathway.

In conclusion, this is the first study to demonstrate the protective role of kallistatin in vascular senescence and aging. Kallistatin's anti‐aging effect is mainly attributed to suppression of oxidative stress by preventing miR‐34a‐mediated inhibition of antioxidant gene expression. This study established an essential role of kallistatin's active site in the regulation of miR‐34a, SIRT1, and eNOS synthesis by interaction with a tyrosine kinase. As kallistatin is an endogenous protein, minimal side effects are expected with kallistatin treatment. Therefore, this study opens a new prospective in kallistatin‐based therapeutic intervention in age‐associated cardiovascular diseases.

## Experimental procedures

### Purification and characterization of recombinant human kallistatins

Recombinant human kallistatin was secreted into serum‐free medium of cultured HEK293T cells, and the cultured medium was concentrated by ammonium sulfate precipitation followed by nickel affinity chromatography as described previously (Chen *et al*., 2000a,b; Li *et al*., 2014). Wild‐type kallistatin, heparin‐binding site mutant kallistatin (K312A/K313A), and active site mutant kallistatin (A377T) were expressed in *E. coli* and purified as described (Chen *et al*., 2000a,b). The purity and identity of human kallistatin were verified by SDS‐PAGE and Western blot using a specific monoclonal antibody (Chao *et al*., 1996; Li *et al*., 2014).

### Isolation and culture of EPCs

EPCs were isolated from human umbilical cord blood as described (Gao *et al*., 2014). The study was approved by the Medical University of South Carolina Human Research (Pro00017277). EPCs were isolated by density gradient centrifugation using Ficoll‐Paque PLUS (GE Healthcare, Waukesha, WI, USA) and cultured in endothelial basal medium 2 with 10% fetal bovine serum and supplements (Lonza, Walkersville, MD, USA).

### β‐Galactosidase staining

EPCs at 80% confluency in 12‐well plates were pre‐incubated with kallistatin (1 μm) for 30 min and then treated with TNF‐α (10 ng mL^−1^) for 6 days. The senescence phenotype was detected using the β‐galactosidase staining kit (Cell Signaling, Danvers, MA, USA). The number of positive senescence‐associated β‐galactosidase (SA‐β‐gal) cells was observed by light microscopy (Olympus CK40, Japan) in 10 randomly chosen low‐power fields (× 200) and expressed as a percentage of counted cells.

### Telomerase activity assay

EPCs were pre‐incubated with or without 1 μm kallistatin for 30 min prior to the addition of 10 ng mL^−1^ TNF‐α for 24 h. Cells were lysed with nondenaturing lysis buffer. Telomerase activity was measured using TRAPEZE RT telomerase detection kit (EMD Millipore, Billerica, MA, USA).

### NADPH oxidase activity assay

The enzymatic activity of NADPH oxidase was assessed by a luminescence assay in the presence of lucigenin (250 μm) and NADPH substrate (100 μm; Sigma, St Louis, MO, USA) as described (Liu *et al*., 2010). Fluorescence intensity was continuously monitored for 15 min with a TD20/20 luminometer. The chemiluminescent signal was corrected by the protein concentration of each sample homogenate.

### Detection of superoxide formation

Cellular ROS generation was detected using the peroxide‐sensitive fluorescent probe 2′,7′‐dichlorodihydrofluorescein diacetate (DCFH‐DA; Sigma) as described (Shen *et al*., 2010). EPCs were seeded in 6‐well plates and were incubated for 30 min with 5 mm DCFH‐DA. EPCs were pretreated with human kallistatin (1 μm) for 30 min before the TNF‐α (10 ng mL^−1^) addition for 30 min. To determine the role of PI3K‐Akt‐eNOS pathway, EPCs were pretreated with PI3K inhibitor LY294003 (5 μm) or iNOS inhibitor L‐NAME (100 μm) for 30 min in prior to kallistatin treatment. To quantify ROS levels, EPCs were seeded onto a 96‐well fluorescence plate and treated as above. Relative fluorescence was measured using the fluorescence plate reader (Biotek, Winooski, VT, USA) at 485‐nm excitation and 535‐nm emission.

Superoxide levels in aortas were determined by fluorescent probe DHE (Shen *et al*., 2010). Briefly, aortic ring segments (8 μm thick) were stained with 3 μm DHE in a light‐protected humidified chamber at 37 °C for 30 min. Images were obtained with a fluorescence microscope (Olympus CK40, Japan).

For ROS staining in *C. elegans,* worms were washed, incubated with 3 μm DHE for 30 min and anesthetized with 5 mm levamisole (Sigma). *Caenorhabditis elegans* were then transferred to glass slides, sealed with 70% glycerol, and imaged with a fluorescence microscope (Olympus CK40, Japan). Fluorescence intensity was analyzed using image j software (National Institutes of Health, Bethesda, MD, USA).

### RNA extraction and real‐time quantitative PCR (Q‐PCR)

Total RNA was extracted using TRIzol as per the manufacturer's instructions. Total RNA was reverse‐transcribed using the High‐Capacity cDNA Reverse Transcription Kit or microRNA Reverse Transcription Kit (Applied Biosystems, Foster City, CA, USA). RT primers for miRNAs were used as follows: U6 for EPCs and mice, has‐miR‐34a and has‐miR‐21 for EPCs, mmu‐miR‐34a‐3p and mmu‐miR‐21 for mice, and U18 and cel‐miR‐34 for *C. elegans*. Real‐time PCR was performed with Taqman Gene Expression Assay kit (Applied Biosystems). Human 18S and U6 were used as internal control genes for quantifying mRNA and miRNA expression in EPCs, respectively. GAPDH and U18 were used as internal control genes for quantifying mRNA and miRNA expression in *C. elegans*, respectively. GAPDH and U6 were used as internal controls for quantifying mRNA and miRNA expression in mice. The following primers were used: 18S (Hs99999901_s1), U6 snRNA (001973), p16^INK4a^ (Hs00923894_m1), hsa‐miR‐34a (002316), eNOS (Hs01574659_m1), SIRT1 (Hs01009006_m1), catalase (Hs00156308_m1), PAI‐1 (Hs01126606_m1), GAPDH (Mm99999915_g1), eNOS (Mm00435217_m1), SIRT1 (Mm00490758_m1), catalase (Mm00437992_m1), U18 (001764), miR‐34 (Ce241995_mat), GAPDH (Ce02425762_m1), sir‐2.1 (Ce02459018_g1), and sod‐3 (Ce02404515_g1). Data were analyzed with 2^−ΔΔCt^ value calculation using control genes for normalization.

### Western blot analysis

Proteins from cell lysates or tissue lysates were separated by a 10% SDS‐polyacrylamide gel electrophoresis and transferred to a nitrocellulose membrane. After being blocked in 7% nonfat milk, protein blots were probed with a primary antibody followed by incubation with a peroxidase‐conjugated secondary antibody. Primary antibodies were rabbit polyclonal anti‐SIRT1, mouse monoclonal anti‐eNOS, mouse monoclonal anti‐β‐actin, and rabbit monoclonal anti‐GAPDH. Chemiluminescence was detected by the ECL‐plus kit (GE Healthcare). Band intensity was quantified by image j software (National Institutes of Health).

### miRNA transfection

For miR‐34a overexpression, EPCs were transfected with 5 pm of miR‐34a mimic or control miRNA (Fisher Scientific, Pittsburgh, PA, USA) with Lipofectamine RNAiMAX reagent (Fisher Scientific) for 12 h according to the manufacturer's protocol.

### Diabetic mouse experiments

All procedures complied with the standards for care and use of animal subjects as stated in the Guide for the Care and Use of Laboratory Animals. The protocol for all animal studies was approved by the Institutional Animal Care and Use Committee at the Medical University of South Carolina. Male wild‐type C57/BL6 mice (7–8 weeks of age) were purchased from Harlan and housed in germ‐free environment. Mice (male, 12‐week‐old) were fasted for 16 h and subjected to 6‐day continuous intraperitoneal injections of streptozotocin (STZ, 60 mg kg^−1^; Sigma) to induce type 1 diabetes. Sodium citrate buffer alone (0.1 m, pH 4.5) was injected in control animals. Mice with blood glucose > 250 mg dL^−1^ were considered diabetic and used in this study. A total of 18 mice were randomly assigned to three groups: control group (*n* = 6), diabetes group (*n* = 6), and diabetes + kallistatin group (20 mg kg^−1^ body weight, *n* = 6). Mice were injected with kallistatin intraperitoneally every 2 days after verification of diabetes. One week after kallistatin injection, thoracic aortas were collected for histological analysis or gene expression analysis.

### *Caenorhabditis elegans* treatments

Wild‐type (N2) *C. elegans,* sir‐2.1 mutant strain VC199 (*ok434*), miR‐34 mutant strain (*gk437*), and SOD‐3::GFP strain CF1553 (*muIs84*) were obtained from the Caenorhabditis Genetics Center (St. Paul, MN). *Caenorhabditis elegans* were cultured and maintained at 25 °C on nematode growth medium (NGM) seeded with *E. coli* OP50. N2 worms were treated with different concentrations of kallistatin (1 or 5 μm) from L4 stage (adult) for 3 days. Total RNA was extracted for real‐time PCR.

### Stress resistance assay

For heat‐shock assay, age‐synchronized N2 L4 worms (*n* = 30) were pre‐incubated with or without kallistatin (1 or 5 μm), and were cultured with daily exchanged fresh medium plates. Two days later, adult worms were exposed at 35 °C for 6 h. The survival of worms was recorded after thermal stress by touching or tapping as monitored under microscope (AmScope). For oxidative stress resistance assay, age‐synchronized N2L4 worms (*n* = 50) were pre‐incubated with or without kallistatin (1 or 5 μm). Next, the three groups were separately transferred to NGM plates containing 2 mm paraquat (Sigma). Survival of the worms was scored daily until the death of last worm.

### Statistical analysis

Data are expressed as means ± SE of three independent experiments. Student *t*‐test and analysis of variants were used to assess differences. Survival curves between treatment groups were compared using the nonparametric log‐rank test. A value of *P *<* *0.05 was considered statistically significant.

## Author contributions

Youming Guo, Pengfei Li, Lee Chao, and Julie Chao conceived and designed the experiments. Youming Guo, Pengfei Li, Zhirong Yang, Lin Gao, and Jingmei Zhang performed the experiments. Youming Guo and Pengfei Li analyzed the data. Youming Guo, Pengfei Li, Eugene Chang, Zhirong Yang, Julie Chao, and Lee Chao contributed reagents/materials/analysis tools. Youming Guo, Pengfei Li, Grant Bledsoe, and Julie Chao wrote the paper.

## Funding

This study was supported by the National Institutes of Health grant HL118516.

## Conflict of interest

None declared.
